# Identification of potential HIV restriction factors by combining evolutionary genomic signatures with functional analyses

**DOI:** 10.1186/s12977-015-0165-5

**Published:** 2015-05-16

**Authors:** Paul J McLaren, Ali Gawanbacht, Nitisha Pyndiah, Christian Krapp, Dominik Hotter, Silvia F Kluge, Nicola Götz, Jessica Heilmann, Katharina Mack, Daniel Sauter, Danielle Thompson, Jérémie Perreaud, Antonio Rausell, Miguel Munoz, Angela Ciuffi, Frank Kirchhoff, Amalio Telenti

**Affiliations:** École Polytechnique Fédérale de Lausanne, 1015 Lausanne, Switzerland; Swiss Institute of Bioinformatics, 1005 Lausanne, Switzerland; Institute of Molecular Virology, Ulm University Medical Center, 89081 Ulm, Germany; Institute of Microbiology, University of Lausanne, 1011 Lausanne, Switzerland; J. Craig Venter Institute, La Jolla, , CA 92037, , USA

**Keywords:** Host restrictions factors, Evolutionary genomics, HIV-1

## Abstract

**Background:**

Known antiretroviral restriction factors are encoded by genes that are under positive selection pressure, induced during HIV-1 infection, up-regulated by interferons, and/or interact with viral proteins. To identify potential novel restriction factors, we performed genome-wide scans for human genes sharing molecular and evolutionary signatures of known restriction factors and tested the anti-HIV-1 activity of the most promising candidates.

**Results:**

Our analyses identified 30 human genes that share characteristics of known restriction factors. Functional analyses of 27 of these candidates showed that over-expression of a strikingly high proportion of them significantly inhibited HIV-1 without causing cytotoxic effects. Five factors (*APOL1, APOL6, CD164, TNFRSF10A*, *TNFRSF10D*) suppressed infectious HIV-1 production in transfected 293T cells by >90% and six additional candidates (*FCGR3A, CD3E, OAS1, GBP5, SPN, IFI16*) achieved this when the virus was lacking intact accessory *vpr*, *vpu* and *nef genes*. Unexpectedly, over-expression of two factors (*IL1A, SP110*) significantly increased infectious HIV-1 production. Mechanistic studies suggest that the newly identified potential restriction factors act at different steps of the viral replication cycle, including proviral transcription and production of viral proteins. Finally, we confirmed that mRNA expression of most of these candidate restriction factors in primary CD4+ T cells is significantly increased by type I interferons.

**Conclusions:**

A limited number of human genes share multiple characteristics of genes encoding for known restriction factors. Most of them display anti-retroviral activity in transient transfection assays and are expressed in primary CD4+ T cells.

**Electronic supplementary material:**

The online version of this article (doi:10.1186/s12977-015-0165-5) contains supplementary material, which is available to authorized users.

## Background

Antiretroviral innate defense genes such as *TRIM5α*, *APOBEC3G, BST2*/*Tetherin,* and *SAMHD1* exhibit characteristic evolutionary signatures of powerful selective pressures reflecting a long-standing evolutionary arms race between the host and viral pathogens [1]. A second common characteristic of these genes is that they are interferon-inducible and differentially expressed during HIV infection [2,3]. Furthermore, they frequently interact directly with viral proteins, either to exert their antiviral activity or because they are targeted by viral antagonists [4-6]. Thus, evolutionary and molecular characteristics, such as positive selection in primate genomes, differential expression during infection, and interaction with viral components might constitute a distinct signature of genes endowed with antiviral activity.

We leveraged the availability of complete genome sequences of several primate species (human, chimpanzee, gorilla, orangutan, macaque, marmoset, tarsier, bushbaby, and mouse lemur) to perform a genome-wide screen for genes carrying the signatures of known host restriction factors. To address this, we examined which human genes that are differentially expressed during HIV-1 infection, and/or encode host factors interacting with viral proteins have also been subject to diversifying selection during primate evolution. Candidates carrying the most promising combined signatures were examined for their effects on different steps of the HIV-1 replication cycle. We emphasized the confirmation of the IFN-induced nature of the candidates, their significant expression in HIV-1 target cells, the efficient reduction of infectious virus production in the over-expression screen, and a particular emphasis on genes that affected the infectiousness of HIV-1 more severely than viral gene expression and/or showed some specificity for the LTR promoter. The combination of bioinformatics criteria with a broad functional screen allowed bringing a large data set of genes to a manageable list of candidates for further analyses. Our results demonstrate that over-expression of a surprisingly high proportion of these genes inhibits infectious HIV-1 production and suggest that the viral accessory proteins Vpr, Vpu and/or Nef may diminish the antiviral effect of some of these cellular factors.

## Results

### Genes that are induced during HIV-1 infection have a distinct evolutionary profile

**Figure 1:**
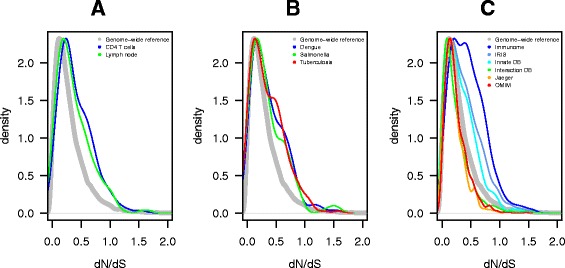
Evolutionary pattern of the protein coding genome in primates. Probability density curves of continuous dN/dS values for genes **(A)** upregulated in CD4+ T cells and in lymph nodes during infection with HIV-1 *in vivo* in humans, **(B)** genes differentially regulated during infection of humans with other pathogens and **(C)** datasets of human innate immunity genes including: an innate immune specific set (Innate DB), genes curated by the Immunogenetic Related Information Source (IRIS) and a manually curated list of immune genes (Immunome), the NCBI HIV interaction database (Interaction DB), the global landscape of HIV-human protein complexes from Jaeger et al. (Jaeger) [15], and of genes associated with Mendelian disorders in OMIM. The kernel smoothed density estimates (density) of dN/dS values for sets of genes is plotted. The height of the curves is relative to the number of genes with the observed dN/dS values. The genome-wide background dN/dS values for 17,755 genes is shown in grey. Statistically significant differences (Kolmogorov-Smirnov statistics *p* ≤ 0.05) were observed for distributions towards greater dN/dS values for genes differentially expressed in CD4+ T cells and lymph nodes during HIV-1 infection, in infection with Dengue virus, *Salmonella typhic* and *tuberculosis*, and for distributions towards smaller dN/dS values for genes involved in HIV-1-human interaction datasets and in the catalog of Mendelian disorders.

To examine the contribution of differences in cellular gene expression to viral control, we have previously generated transcriptome data from CD4+ T cells of untreated HIV-1 infected individuals with different viral loads [3,7]. Additional gene expression data were obtained from published sources on lymph nodes during HIV-1 infection [8]. We also assessed the evidence for evolutionary pressure on all genes by comparing human gene sequences to those of eight additional simian and prosimian species (see methods) and calculated a gene-wide ratio of non-synonymous (dN) to synonymous (dS) substitutions (gene dN/dS). We found that genes whose expression is positively correlated to viral load in CD4+ T cells (n = 180) or induced in lymph nodes (n = 360) of HIV-1 infected persons had higher dN/dS values than the genome-wide median for primates (CD4+ T cell gene set, dN/dS 0.25 vs 0.18, *p* <10^−5^, and lymph node gene set dN/dS 0.28 vs 0.18, *p* <10^−5^) (Figure 1A). Genes with dN/dS values inflated above the genome-wide reference could either be evolving under more neutral selection, or could have within them certain codons evolving under positive selection that bring up the gene-wide dN/dS value. Across these expression datasets, 30 genes up-regulated during HIV-1 infection were under positive selection (dN/dS >1).

We next examined whether such an evolutionary pattern is also observed in other human infections [9-12]. Compared with the dN/dS genome median of 0.18, genes that are differentially expressed during Dengue virus infection (n = 158), *salmonellosis* (n = 205), or active *tuberculosis* (n = 251) generally had higher dN/dS values (Dengue, 0.22, *p* = 0.02; *salmonellosis* 0.20, *p* = 0.04; and *tuberculosis* 0.23, *p* = 2×10^−5^) (Figure 1B). A similar pattern was identified for three curated sets of innate immunity genes (http://www.innatedb.ca; n = 1714) that displayed a statistically significant shift to high dN/dS values (all p < 2×10^−4^) (Figure 1C). Altogether, these results demonstrate that genes associated with infection and immune response show distinctly higher dN/dS values in primates. Although a higher dN/dS value could be indicative of relaxed constraint rather than positive selection [13], immune-related genes have consistently been observed to evolve with higher dN/dS values and show evidence for codon-specific positive selection [14].

We then extended the analysis to genes described in the NCBI HIV-1 protein interaction database (n = 1251) and in the global landscape of HIV-human protein complexes (n = 350) [15]. These sets of genes had a significantly lower median dN/dS than the genome-wide median for primates (0.14 and 0.13 vs 0.18, respectively, both *p* <10^−5^, Figure 1C). The shift to conservation is comparable to that of other characteristically conserved genes such as those of the OMIM Mendelian disease database (n = 1289) (Figure 1C). This most likely reflects the fact that HIV has to interact with numerous well-conserved cellular factors (“HIV dependency factors”) to complete its replication cycle. Although the two HIV interaction datasets were more conserved, 35 genes had dN/dS >1. As indicated below, some genes under positive selection were common to both the expression and the interaction datasets.

### Selection of 30 candidate restriction factors for experimental validation

**Figure 2:**
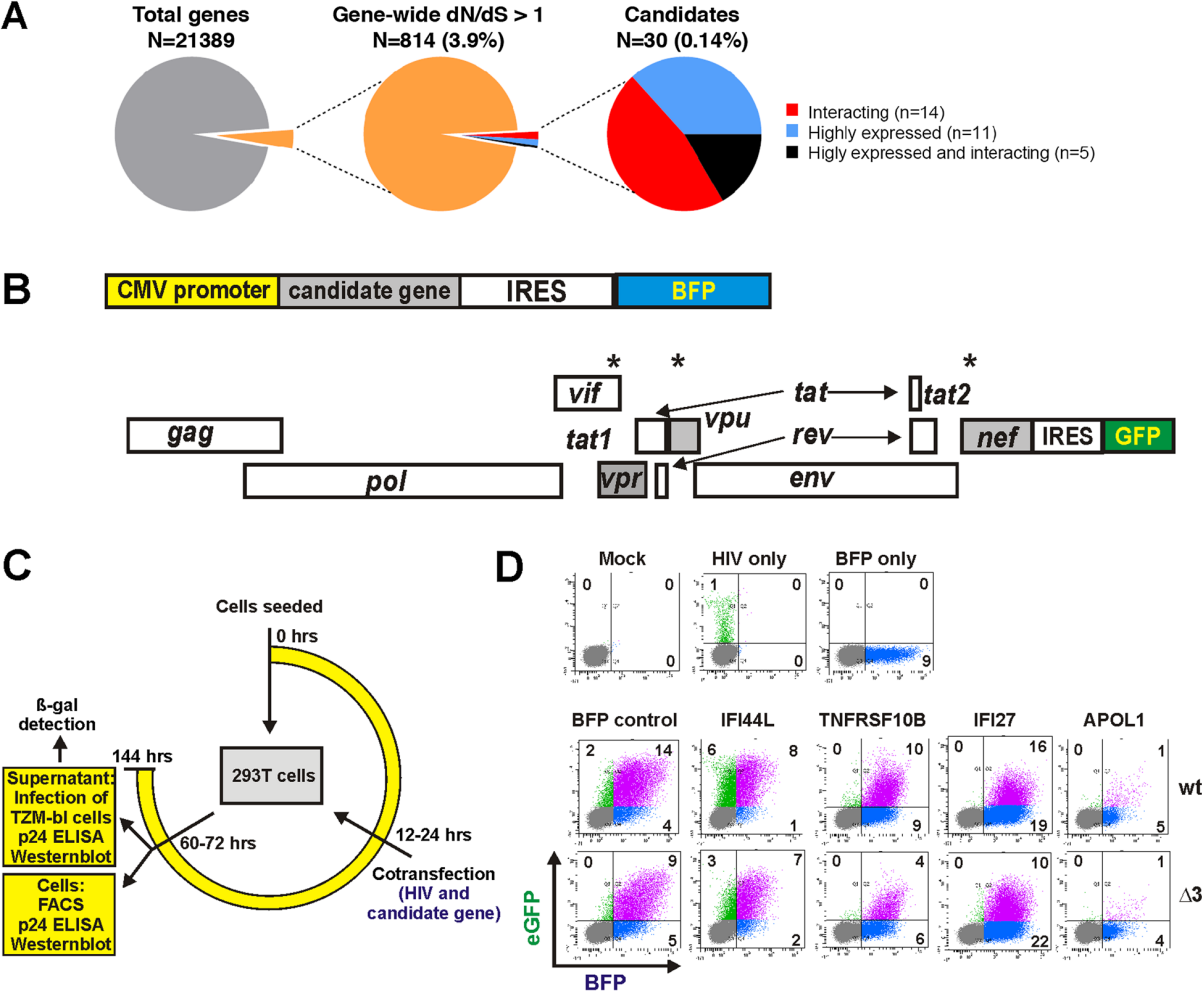
Experimental setup of the screen and the different measurements involved. **(A)** In orthogonal screens, 841 human genes were identified to be under positive selection, of which only 30 were retained as candidates (11 were upregulated during HIV-1 infection, 14 reported to interact with viral proteins, and 5 fulfilled both criteria). **(B)** Schematic presentation of the bi-cistronic constructs coexpressing the gene candidate together with the blue fluorescent protein (BFP) under the control of a CMV promoter and the HIV-1 NL4-3-based IRES-eGFP proviral reporter construct. The position of premature stop codons in the *vpr*, *vpu* and *nef* genes of the Δ3 derivative are indicated by stars. **(C)** Overview of the experimental outline to determine the effect of cotransfected gene candidates on early viral gene expression (detection of eGFP by FACS) and late viral gene expression (cell-associated p24 antigen ELISA), virion release (cell-free p24 antigen ELISA) and viral infectivity (TZM-bl reporter assay). **(D)** Examples of primary FACS data. 293T cells were transfected with the indicated proviral constructs and expression vectors and analyzed by flow cytometry 48 h later.

Our initial evolutionary scan identified 841 genes (out of a total of 21,389) with a dN/dS ratio > 1 (Figure 2A). We next evaluated in detail the functional profile of this subset by assessing their level of upregulation during HIV-1 infection *in vivo*, and/or evidence for interaction with viral proteins (see Methods) resulting in an initial shortlist of 57 genes (Additional file 1: Table S1). To confidently assess the genes' evolutionary signatures, we used the codon-level analysis package (codeml) in the Phylogenetic Analysis Using Maximum Likelihood (PAML [16]) software on curated multiple alignments of primate genes (see Methods). This method averages per-codon dN/dS estimates across a gene and assesses the evidence for codon-level selection using maximum likelihood estimation. Of the initial 57 candidates, we selected 30 genes for functional analyses based on significant evidence of positive selection at one or more amino acid sites (Additional file 1: Table S1). Eleven of these 30 candidate genes were chosen because they are upregulated during HIV-1 infection, 14 have been shown to interact with viral proteins, and five fulfill both criteria (Figure 2A). As a proof-of-principle, *TRIM5α* and *APOBEC3G* were identified in the screen. Consistent with previous reports [17,18], *BST2* and *SAMHD1* reached dN/dS ratios of 0.78 and 0.49, respectively; therefore, they were not shortlisted on the basis of global dN/dS ratios, although they displayed codon-specific positive selection. To also consider factors displaying only codon-specific positive selection, we additionally performed a second screen and ranking approach that are described below.

### Cell based assays for assessment of restriction

To examine possible antiviral effects of the candidate genes, we developed cell-based assays to determine their impact on HIV-1 gene expression, virus production and viral infectivity. A total of 27 candidate genes Additional file 1: Table S1 were cloned into a bi-cistronic vector coexpressing the gene of interest and the blue fluorescent protein (BFP) via an internal ribosome entry site (IRES) (Figure 2B). Three of the 30 genes (*PARP9, SGOL2, SPAG5*) could not be PCR amplified and cloned from cDNA of primary blood cells. 293T cells were cotransfected with the gene expression constructs and HIV-1 NL4-3-based proviral IRES/eGFP constructs. The latter express Nef and the enhanced version of the green fluorescent protein (eGFP) from the same viral RNAs [19]. Thus, the green fluorescence intensity is an indicator of early viral gene expression. To identify possible antagonistic effects of the viral Vpr, Vpu and Nef proteins on the candidate gene products, we screened them for inhibition of a wild-type (wt) NL4-3-based HIV-1 construct and an otherwise isogenic derivate thereof (named Δ3) containing defects in these three accessory genes (Figure 2B). The effect of candidate genes on early LTR-driven gene expression was quantified by fluorescence-activated cell sorting (FACS) at 36 to 48 hours post-transfection (Figure 2C). To monitor the expression of late viral gene products, virus release and infectivity of progeny virions, we assessed the quantity of cell-associated and cell-free p24 capsid antigen and the infectivity of the viral supernatants (Figure 2C). The FACS-based assay allowed us to readily determine the percentage of cells expressing the proviral HIV-1 genome and the cellular candidate genes both alone and in combination (Figure 2D). Importantly, none of the 27 candidate genes caused significant cytotoxic effects or reduced the metabolic activity of the cells under the experimental conditions used to determine their antiviral activity (Additional file 2: Figure S1).

### A high proportion of candidate genes display anti-HIV-1 activity

**Figure 3:**
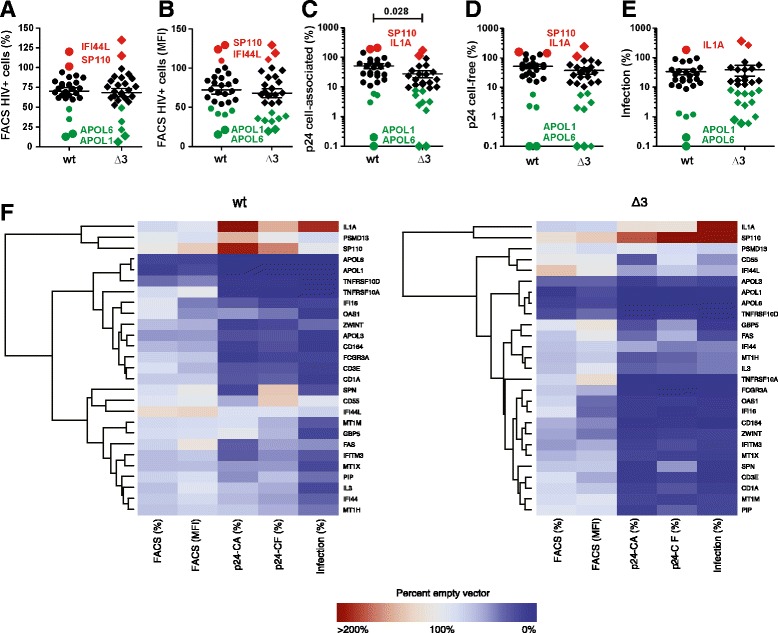
Identification of candidate genes that affect HIV-1 replication. **(A)** HIV-1-expressing (eGFP+) cells and **(B)** mean eGFP fluorescence intensities in the presence of over-expressed gene candidates at 48 h post transfection. **(C)** Levels of infectious HIV-1 determined by infection of TZM-bl indicator cells with the culture supernatant of transfected 293T cells. **(D, E)** cell-free (CF) and cell-associated (CA) p24 antigen levels from 293T cell cultures cotransfected with wt or *vpr*, *vpu* and *nef* defective (Δ3) HIV-1 constructs and gene candidate expression vectors. All panels provide values relative to those measured in 293T cultures cotransfected with the wt and Δ3 proviral HIV-1 constructs and the control vector expressing BFP only (100%). Each symbol represents the average value obtained from three independent experiments for each of the gene candidates analyzed. All candidates that reduced the values <50% in the FACS-based and <10% in the remaining assays are highlighted in green and selected genes that showed enhancing effect are indicated in red. **(F)** Unsupervised clustering of the different readouts allows inspection of similar activities across genes, and of the correlation between the various steps, for wt and Δ3 HIV-1.

We found that most candidate genes reduced the frequency of HIV-1-expressing (eGFP+) cells, on average by about 30% (Figure 3A, Additional file 2: Figure S2 and Additional file 1: Table S1). Expression of four candidate genes (*APOL1, APOL3, APOL6* and *TNFRSF10D*) achieved more than 50% inhibition, whereas two genes (*IFI44L*, *SP110*) slightly increased the frequency of eGFP+ cells. The effects of the candidate genes on wt and Δ3 HIV-1 constructs in the FACS-based assay correlated significantly (R^2^ = 0.93; p < 0.0001) (Additional file 2: Figure S3A). This was expected because defects in the *vpr*, *vpu* and *nef* genes should not affect early viral gene expression upon transfection of proviral HIV-1 constructs.

The FACS-based assay also provides an indication for the strength of viral gene expression based on the eGFP mean fluorescence intensity (MFI) since Nef and eGFP are expressed from the same double-spliced bi-cistronic RNA. We found that most candidate genes suppressed LTR-dependent eGFP expression (Figure 3B) and observed a significant but imperfect correlation (R^2^ = 0.57, p < 0.0001) between the effects of the candidate genes on the percentages of eGFP+ cells and the MFIs of eGFP (Additional file 2: Figure S3B). In addition to the four factors mentioned above (*APOL1, APOL3, APOL6* and *TNFRSF10D*), two additional candidates (*OAS1* and *IFI16*) reduced the MFI of eGFP by more than 50% (Additional file 2: Figure S2 and Additional file 1: Table S1).

On average, the candidate genes suppressed the levels of intracellular p24 by 49.1% (Figure 3C) and cell-free p24 production by 48.2% (Figure 3D) for the wt virus. All candidate genes that reduced p24 antigen expression and production in cells infected with the wt HIV-1 construct also inhibited the Δ3 virus but the effects were often stronger, i.e. 72.5% (p = 0.028) reduction of cell-associated and 63.8% reduction of cell-free p24 production (Figures 3C and 3D). A total of eight cellular factors (*APOL1, APOL6, TNFRSF10A, TNFRSF10D, IFI16, CD164, FCGR3A* and *TRIM5α*) caused >90% reduction of p24 release by the Δ3 virus (Additional file 2: Figure S2). We found a significant correlation between the quantities of cell-free and cell-associated p24 antigen (R^2^ = 0.62 p < 0.0001) (Additional file 2: Figure S3) and none of the 27 factors analyzed specifically suppressed virion assembly and/or release in the transient transfection assay.

Altogether, over-expression of more than half (15 of 27) of the candidate genes reduced the production of infectious wt HIV-1 by more than 75% and five factors (*APOL1, APOL6, TNFRSF10A, TNFRSF10D* and *CD164)* achieved >90% inhibition (Figure 3E, Additional file 2: Figure S2 and Additional file 1: Table S1). All candidate factors that suppressed the production of infectious wt HIV-1 also inhibited the Δ3 HIV-1 construct (Additional file 2: Figure S3A). However, the inhibitory effects on the Δ3 HIV-1 mutant were frequently even more pronounced. In addition to the candidates listed above, six additional cellular genes (*OAS1, CD3E, GBP5, FCGR3A, IFI16*, and *SPN*) achieved >90% reduction of infectious HIV-1 production if the virus was lacking intact *vpr*, *vpu* and *nef* genes (Figure 3E, Additional file 2: Figure S2 and Additional file 1: Table S1). Two factors (*IL1A* and *SP110*) enhanced the production of infectious HIV-1. The enhancing effect was particularly pronounced for the Δ3 HIV-1 construct (Figure 3E, Additional file 2: Figure S2 and Additional file 1: Table S1). Altogether, the levels of viral infectivity and cell-free p24 correlated well (R^2^ = 0.66; p < 0.0001) although the effects on the former were frequently more pronounced (Additional file 2: Figure S3B). Some factors, such as *GBP5* and *SPN* reduced virion infectivity more severely (16.4% and 23.9%) than the release of p24 antigen (59.9% and 132.5%) (Figure 3F, Additional file 2: Figure S2 and Additional file 1: Table S1) and may thus impair the infectiousness of progeny virions*.*

To obtain first insights into the possible mechanisms underlying the inhibitory effects, we examined whether the reduced levels of eGFP+ (HIV-1-expressing) cells correlate with the levels of infectious virus production (Figure 3F). We found a significant but imperfect correlation (R^2^ = 0.23; p = 0.0094) (Additional file 2: Figure S3B) suggesting that reduction of viral gene expression or translation is important but not the only inhibitory mechanisms. For example, the *TNFRSF10* genes reduced eGFP expression in the FACS-based assay substantially less effectively than *APOL1, APOL3* and *APOL6* but achieved a similar reduction in infectious virus production (Figure 3F, Additional file 2: Figure S2 and Additional file 1: Table S1).

### Candidate restriction factors inhibit HIV-1 in a dose-dependent manner

**Figure 4:**
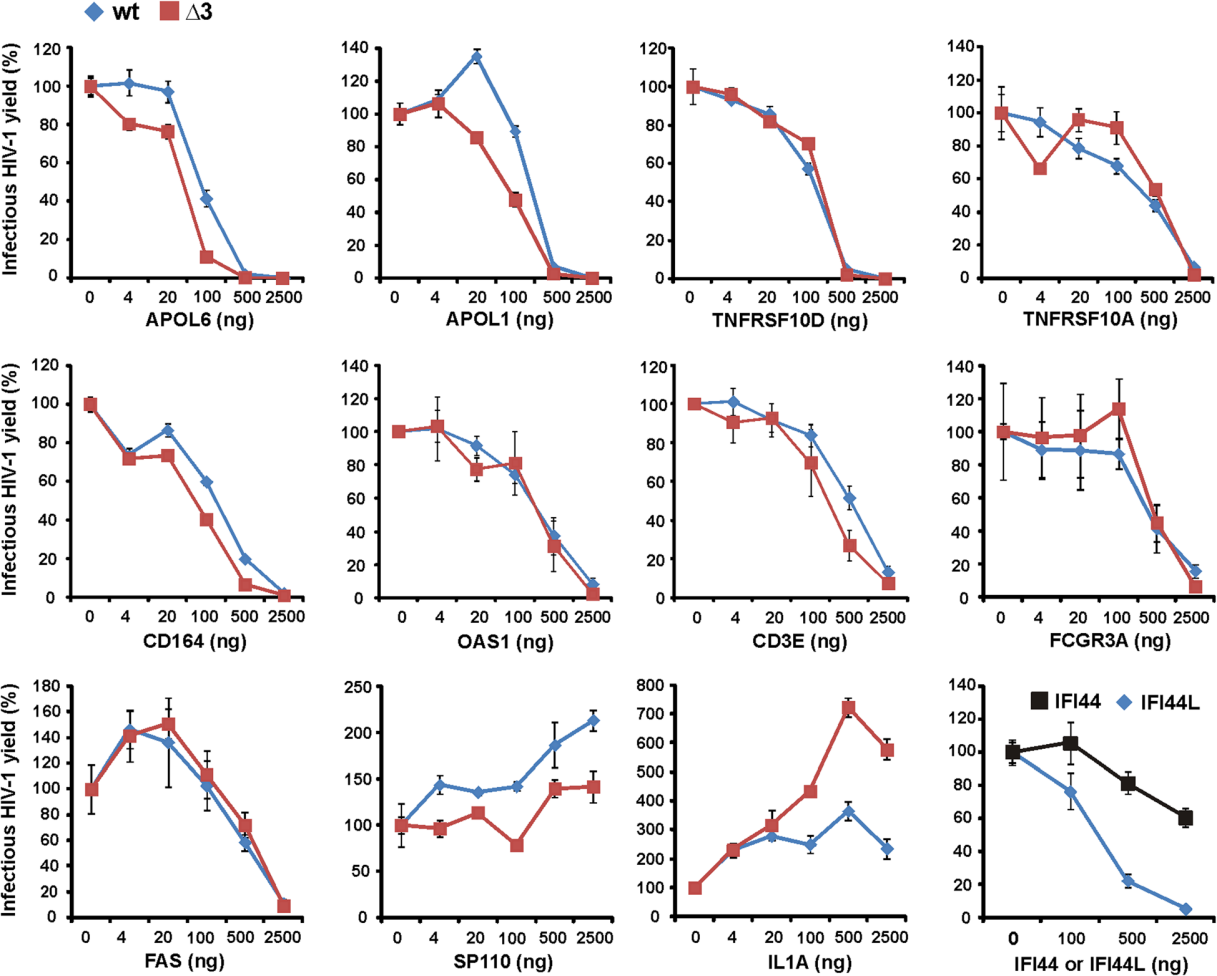
Titration of the effects of the gene candidates on infectious HIV-1 production. Virus release from 293T cells following transfection with 5 μg of wt or Δ3 proviral NL4-3 constructs and varying amounts of plasmids expressing the indicated candidate genes. The total quantity of transfected DNA was adjusted to 7.5 μg with empty vector. Infectious virus yield was determined by infection of TZM-bl indicator cells and is shown as percentage of that detected in the absence of candidate gene expression. Values represent averages (±SD) derived from triplicate infections and the results were confirmed in an independent experiment.

To further verify the antiretroviral activities, we next measured infectious virus yields from 293 T cells after cotransfection with fixed quantities of wt or Δ3 HIV-1 NL4-3 proviral vectors and increasing doses of plasmids expressing 13 selected candidate genes. These titration experiments confirmed that *APOL1, APOL6, TNFRSF10A, TNFRSF10D, CD164, OAS1, CD3E* and *FCGR3A* inhibit HIV-1 in a dose-dependent manner (Figure 4). The titration analyses also showed that some genes (*APOL1*, *APOL6*, *CD164* and *CD3E*) reduced the yield of infectious wt HIV-1 slightly less efficiently than of the Δ3 virus. Interestingly, *FAS* generally increased infectious HIV-1 production at low expression levels but significantly reduced it at the highest dose of expression plasmids (Figure 4). We also verified that *SP110* and *IL1A* increased the infectious virus yield. Strikingly, *IL1A* increased infectious virus yield by up to 3.5-fold for the wt and even up to 7-fold for the Δ3 construct. Finally, we examined the effects of the interferon-induced *IFI44* and *IFI44L*. Notably, the latter reduced infectious HIV-1 yield more efficiently (Figure 4) although both genes share a significant degree of sequence homology. Altogether, the titration experiments confirmed that many of the newly identified candidate restriction factors suppress infectious HIV-1 production in transiently transfected 293T cells.

### A high proportion of candidates affect the activity of viral promoters

**Figure 5:**
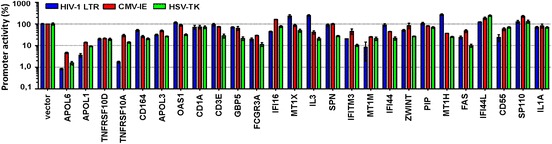
Effect of candidate gene expression on viral promoter activity. Effect of the indicated candidates on the activity of the HIV-1 LTR in the presence of Tat and the CMV-IE and HSV-TK promoters. All data were normalized to the BFP only control construct (100%) and represent average values (±SEM) derived from three transfections.

The FACS-based assays suggested that some of the candidate factors may reduce the transcription of viral genes. To examine the effect of the candidate factors on HIV-1 promoter activity, we cotransfected 293T cells with a firefly luciferase reporter vector controlled by the HIV-1 LTR, plasmids coexpressing the candidate genes and BFP, and a construct expressing the NL4-3 Tat protein. For comparison, we also analyzed the effects on the herpes simplex virus thymidine kinase (HSV-TK) and cytomegalovirus immediate early (CMV-IE) promoters. Many candidate factors, particularly *APOL* family members and *FCGR3A* encoding the low affinity immunoglobulin gamma Fc region receptor III-A, efficiently suppressed HIV-1 LTR activity (Figure 5). However, the effects were not specific for HIV-1 since these candidate factors also inhibited the activity of the CMV-IE and HSV-TK promoters. Perhaps more notably, several candidate genes, e.g. *CD1A, CD3E, GBP5 and SPN* had little if any effect on viral LTR activity (Figure 5) but still efficiently prevented the production of infectious HIV-1 (Additional file 2: Figure S2). Thus, the results confirmed that reduced viral gene expression is an important but not the only inhibitory mechanism.

### A high proportion of candidate genes are interferon-inducible in CD4+ T cells

**Figure 6:**
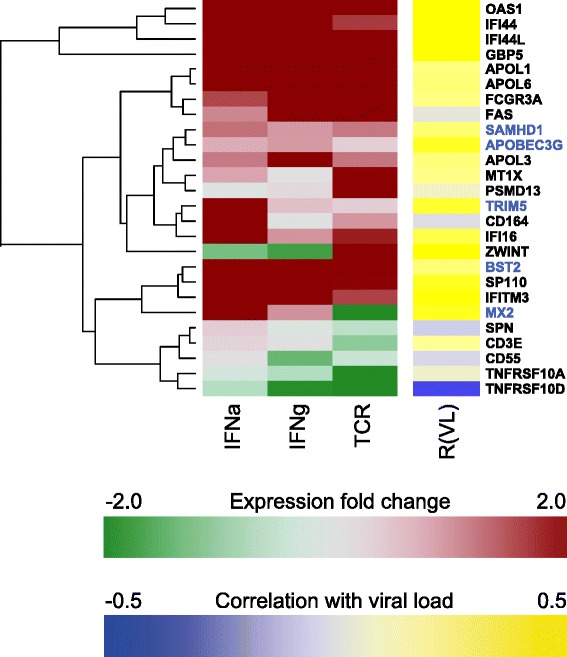
Heat map of expression after stimulation of known HIV-1 restriction factors and gene candidates through the screening approach. On the left, CD4+ T cells from healthy donors were left unstimulated or stimulated with IFNα, IFNγ or αCD3/CD28. Expression levels of known restriction factors (blue), and factors identified in the screen (black) were clustered using complete hierarchical clustering. For comparison, the right panel depicts the correlation between gene expression in CD4+ T cells of HIV infected donors with setpoint viral load (R(VL)). Correlation values were taken from [3]. Positive R(VL) values (yellow) indicate an increased gene expression correlates with an increase in patient viral load (log_10_ RNA copies/ml).

The genome-wide screen used gene expression data from *in vivo* studies in HIV-positive individuals where gene expression was correlated to patient viral load [3,7]. To better dissect the general agreement between *in vivo* data with actual interferon inducibility, we assessed the response of primary CD4+ T cells from healthy donors to stimulation with interferon alpha, interferon gamma and to cell activation via the T cell receptor (TCR). The analysis identified a general agreement between *in vivo* data and interferon alpha gene induction, with the majority of the genes being strongly up-regulated by stimulation (in healthy donors) and by viral replication in HIV+ patients (Figure 6). These data confirm that the *in vivo* response to HIV infection is greatly driven by the interferon response, although other drivers of immune activation, as exemplified here by TCR stimulation, share parts of the signature. Thus, many of the candidate genes identified in the present study are true interferon-stimulated genes (ISGs) as shown by *in vivo* and *in vitro* data and expressed in the main target cells of HIV-1 in infected humans.

### Relaxed screening approach ranks genes with potential antiviral activity

The first screening imposed a hard threshold for inclusion (dN/dS > 1) to identify genes under (global) positive selection pressure. Use of this global gene metric is a useful link to previous literature; however, tools such as PAML that assess local signatures of selective pressure and have built-in statistics are becoming a preferred approach. In particular, genes of some known restriction factors (e.g. *BST2* and *SAMHD1*) just show signatures of codon-specific (local) instead of global positive section pressure. Thus, we explored a second genome-wide approach to rank genes using several metrics based on characteristics of innate immunity genes [20]. We initially considered eight parameters associated with innate immunity genes: dN/dS value, codon-specific positive selection (PAML), measurements of cross species adaptive evolution, burden of synonymous, missense and non-sense variation, intolerance to functional variation and number of paralogs, as well as the *in vivo* gene expression response to HIV-1 infection. We trained a model using the set of known HIV restriction factors (*APOBEC3G, BST2/Tetherin, MX2, SAMHD1 and TRIM5α*). Gene parameters were iteratively included/excluded, and the final set of parameters was selected such that the sum of the ranks across the known genes was minimized (see Methods). We present in Additional file 3: Table S2 the top 200 ranked genes. This is a resource that avoids the strict cutoff criteria of dN/dS >1 to accommodate a more extensive set of gene candidates. Known restriction factors *APOBEC3G*, *TRIM5α*, *MX2*, *SAMHD1* and *BST2* were ranked 2, 5, 125, 277 and 379 among 15,052 protein-coding genes respectively in the final model. Overall, 20 of the 30 candidates identified in the initial screen also ranked above the 95 percentile for the genome in the second approach, and all but one (*TNFRSF10D*) ranked above the 75 percentile.

## Discussion

In the present study, we utilized features of known antiviral host restriction factors [21], such as evolutionary signatures of positive selection, expression during HIV infection, and interaction with viral proteins, to identify novel cellular factors that inhibit HIV-1. Initially, we performed a canonical genome-wide screen revealing that only a small proportion of the genome (841 of 21389 genes; 3.9%) shows evidence for positive selection in primates (dN/dS > 1). Next, we examined which of these genes are significantly upregulated during HIV-1 infection *in vivo* and/or encode for cellular factors known to interact with HIV-1 proteins. Based on these selection criteria 30 candidate genes were selected for closer examination and a strikingly high proportion of them suppressed infectious HIV-1 production in transfected 293T cells. To consider the fact that known restriction factors, such as APOBEC3G, BST2 and SAMHD1, show site-specific rather than global evidence for positive selection, we conducted a second screen with more flexible modeling on features of known HIV restriction factors to rank genes without imposing strict thresholds for calling a candidate. All five known restriction factors (APOBEC3G, TRIM5α, MX2, SAMHD1 and BST2) ranked within the top 2.5% of all protein-coding genes examined (average 1.0%). The analysis extends previous work from our group [17] and work of Meyerson et al. [22] that searched for signatures of positive selection among previously described HIV host factor genes. The current genome-wide analyses allowed identification of additional genes sharing characteristics of known restriction factors, a useful basis for the identification of novel intrinsic antiretroviral cellular factors.

To evaluate possible antiviral effects of the selected genes, we determined their impact on HIV-1 gene expression, virus production and viral infectivity. A set of 27 genes encoding cellular proteins not previously implicated as HIV-1 restriction factors were amplified from primary human blood cells and cloned into a bi-cistronic vector coexpressing the gene candidate BFP. Over-expression of a striking proportion of candidate genes (16 of 27; 59%) reduced the production of infectious wt HIV-1 particles by more than 75% without causing cytotoxic effects and this number was even higher in the absence of intact *vpr*, *vpu* and *nef* genes (20 of 27; 74%). For example, genes of the apolipoprotein L gene family (*APOL1, APOL3, APOL6*) were generally very potent inhibitors. All members of this gene family have rapidly evolved in primates [23], and APOL1 has been shown to confer immunity to *Trypanosoma brucei* that can be countered by a trypanosome-encoded antagonist [24]. Members of the TNF-receptor superfamily (*TNFRSF10A* and *10D*) were also powerful inhibitors. Notably, *TNFRSF10A* was also identified as one of the most potent anti-HIV-1 factors among 380 type I interferon-stimulated genes in a previous study [25]. Another potent inhibitor of HIV-1 was *CD164* (also known as endolyn) a cell adhesion molecule that interacts with CXCR4 [26]. Furthermore, the high activity of *OAS1* is noteworthy because this interferon-induced factor is known to generate 2',5'-oligoadenylates which activate RNase L thus causing viral RNA degradation and inhibition of viral replication [27]. Notably, *OAS1* affected the percentage of HIV-1 infected (eGFP+) cells less severely than the mean fluorescence intensity of eGFP expression, which would be expected in the case of RNase L mediated viral RNA degradation. A similar phenotype was observed for *IFI16*, which is involved in transcriptional regulation and plays a role in the sensing of intracellular viral DNA [28,29]. More recently, IFI16 has been reported as a key player in inducing pyroptosis in abortively infected quiescent CD4+ T cells, thereby contributing to the massive depletion of CD4+ T cells observed during HIV infection [30,31]. Accumulating evidence suggest that restriction factors, such as TRIM5α [32] and BST2/tetherin [33-35] may also act as immune sensors and direct anti-HIV effects of IFI16 warrant further study. Conversely, some genes that displayed anti-HIV activity are known to be involved in signaling, both in various cancers (*CD164, TNFRSF10A/D, ZWINT, MT1X, IL3*) and immune or T cell activation (*GBP5, CD1A*). Thus, these genes may not inhibit HIV-1 directly but could also induce cellular responses associated with the expression of other innate immunity factors. This possibility is in agreement with our observation that some candidate factors inhibited various viral promoters. It is also noteworthy, however, that 293T cells lack many intact immune signaling pathways and did not express detectable levels of interferon after transfection with constructs expressing the various candidate factors. Further studies will be necessary to clearly distinguish between direct and indirect anti-HIV effects.

Two of the 27 cellular genes examined (*SP110* and *IL1A*) enhanced production of infectious HIV-1. *SP110* is part of a leukocyte-specific nuclear body component that may also play a role in the regulation of gene transcription. Mutations in *SP110* are associated with immunodeficiency [36]. It has been reported that this factor stabilizes EBV mRNAs and enhances lytic viral gene expression [37]. The strongest enhancing effects on infectious virus production (up to 7-fold in dose-dependency studies) were observed for *IL1A*. This cytokine plays a key role in inflammation and the regulation of the immune response. It has been reported that HIV-1-exposed seronegative sex workers show reduced levels of *IL1A* expression in their genital mucosa [38]. Thus, the present results confirm and expand previous data supporting that these cellular factors may enhance viral infection and clearly warrant further investigation.

In most cases, the effect of the candidate restriction genes in the FACS-based assays, intra- and extracellular p24 levels and infectious virus production correlated with one another, suggesting that reduced viral gene expression and/or translation are responsible for the inhibitory activity of the cellular factors rather than effects on virus release or virion infectivity. In support of this, preliminary data using LTR-luciferase reporter constructs suggest that many candidates suppress LTR activity (Figure 5). Nonetheless, our results also provide evidence that some candidate genes target different steps of the viral life cycle. For example, the guanylate-binding protein 5 (*GBP5*) and the mucin-like factor *SPN* (also known as leukosialin, sialophorin or CD43) reduced viral infectivity more severely than the levels of cell-free p24 antigen and may thus specifically reduce virion infectivity*.GBP5* is a member of the dynamin superfamily of GTPases, which have been implicated in protection against various pathogens [39,40]. Another factor (*IFITM3*), that was identified by our screen and reduced infectious virus production, was recently shown to be incorporated into HIV-1 virions and to impair their infectiousness [41,42]. *SPN* regulates CD4+ T cell proliferation and migration and shows reduced expression and aberrant glycosylation in HIV-1-infected individuals [43,44]. Thus, it is conceivable that *GBP5, SPN* and *IFITM3* may affect virion infectivity although the underlying molecular mechanisms of the former two remain to be determined.

Some known host restriction factors, i.e. APOBEC3G, Tetherin and SAMHD1, were initially discovered because they are counteracted by viral accessory proteins [4,5]. We found that the absence of intact *vpr*, *vpu* and *nef* genes increased the inhibitory effects of several candidate factors (e.g. *APOL1*, *APOL6, CD164, GBP5, SPN, CD3E, ZWINT* and *PIP*) on p24 and infectious virus production. However, the differences were generally modest because even wt HIV-1 was efficiently inhibited at high expression levels of the candidates. Notably, over-expression of known restriction factors, such as tetherin, can also overcome the viral antagonists. Thus, it remains to be clarified whether the lower intrinsic infectiousness of the Δ3 HIV-1 construct compared to the wt virus or specific antagonistic effects of the accessory Vpr, Vpu and/or Nef are responsible for the observed differences in susceptibility to inhibition by some candidate factors.

Not all recognized restriction factors would have been identified under the criteria of the evolutionary genetic screen because of the criteria that imposed a dN/dS > 1. We could however demonstrate that alternative approaches that model several characteristics of innate immunity genes ranked all known restriction factors and most of the novel candidates among the top 5% genes in the genome. Overall, genes that emerged from the screen were strongly induced by interferon alpha. These results indicate that they are *bona fide* interferon-stimulated genes (ISGs) that are possibly endowed with antiviral activity. Our ongoing studies aim to further define the antiviral mechanisms of the most potent and specific candidate genes as well as their possible interaction with the accessory viral proteins.

In summary, we show that the number of cellular genes showing evolutionary and functional characteristics similar to known host restriction factors is limited. Interferon treatment induced expression of most of these genes in CD4+ T cells and over-expression of a strikingly high proportion of them in transfected 293T cells inhibited the production of infectious HIV-1. These results are consistent with the observation that interferon administration, likely through the action of yet to be characterized interferon stimulated genes, inhibits HIV and related primate lentiviruses [45,46]. The underlying mechanisms and the relevance of these potential restriction factors to the control of HIV-1 replication in relevant viral target cells and *in vivo* remain elusive and further characterization of these genes may improve our understanding of cellular defense mechanisms.

## Experimental Procedures

### Expression and protein interaction datasets

We used transcriptome data from purified CD4+ T cells from 128 HIV-1 infected seroconverting individuals representing the complete range of viral setpoint [3] using Illumina Human HT-12 V3 BeadChip arrays (Illumina). Patient spVL was calculated as the average of all log10 transformed viral load measurements (RNA copies/ml) after the acute phase of infection and prior to disease progression (CD4 < 350 cells/ml) or the initiation of ART as described [47].

Association analysis was performed regressing expression level of each gene on patient spVL controlling for gender, age, CD4+ T cell viability and batch effect. In addition, we compiled data from transcriptome analysis of other active infections that used comparable genome-wide technologies (Illumina, Affymetrix and other arrays containing >20,000 human transcripts): the transcriptome of lymph nodes of HIV-infected individuals [8], and expression analyses of peripheral blood from individuals with tuberculosis [9], typhoid fever [10], and dengue [11,12]. Based on the HIV host transcriptome studies, we defined post-hoc a HIV-1 expressed gene set that included 527 genes upregulated during *in vivo* infection. The NCBI HIV-1-human protein interaction database (www.ncbi.nlm.nih.gov/RefSeq/ HIVInteractions/) and the “global landscape of HIV-human protein complexes” [15] were used to evaluate interactions between HIV-1 and cellular factors. InnateDB (www.innatedb.ca), a publicly available database of the genes, proteins, experimentally-verified interactions and signaling pathways involved in the innate immune response was included to evaluate the general profile of genes participating in the response to microbial infection. Genes were extracted from the OMIM Gene Map (www.ncbi.nlm.nih.gov/sites/entrez?db=omim), and a dedicated set was created by including genes associated with a disease and C = confirmed - observed in at least two laboratories or in several families.

### Codon alignments of primate orthologous genes

Genome-wide codon alignments of orthologous genes for up to nine simian and prosimian species (human, chimpanzee, gorilla, orangutan, macaque, marmoset, tarsier, bushbaby, and mouse lemur) were collected from Ensembl v57. First, one-to-one orthologous primate gene trees were gathered from the Ensembl Compara database by extracting the sub-tree corresponding to all ‘one2one’ primate orthologs of each human gene, as annotated by Compara’s gene tree reconciliation and homology annotation method [48]. These one-to-one primate gene trees, which included branch lengths in units of expected nucleotide substitutions per site, were used to guide the rest of the analysis.

It has been shown that errors in sequencing, annotation and alignment can lead to excessive false positives in downstream evolutionary analyses [49]. As a result, efforts were made to avoid including possibly erroneously sequenced or aligned bases in our dataset. Comparison between Compara’s protein-based alignments and its DNA-based alignments showed that the protein-based alignments often contained missing or alternatively-spliced exons in otherwise highly similar primate orthologs, leading to stretches of erroneously-aligned bases and inflated estimates of evolutionary rates and dN/dS (data not shown). In order to avoid such errors, we gathered human-flattened genomic alignments by using exon coordinates from the human Consensus Coding Sequence (CCDS) transcript, or if no CCDS transcript was available, the longest human protein-coding transcript, to extract all available aligned primate DNA sequences from the ‘12 eutherian mammals’ DNA-based multiple genomic alignments from Ensembl's Enredo-Pecan-Ortheus pipeline [50]. Low-quality genomic sequence was excluded by masking any nucleotides with a PHRED (or PHRED-equivalent) quality score below 30 (corresponding to an error rate of 1 in 1,000) from the chimpanzee, orangutan, macaque, marmoset, tarsier, bushbaby and mouse lemur source genomes with ‘N’ characters; genome quality scores were downloaded from the originating institutions for each genome assembly used in Ensembl v57. Finally, sequences in the generated alignments were cleaned in preparation for codon model analysis by masking out any frameshifting indels (which within these closely-related primates are more likely to be the result of sequencing error than true biological indels) and premature stop codons with ‘N’ characters. The precision of the genome-wide values of dN/dS was assessed by contrasting the estimates with those from 126 curated alignments [17].

The codeml program from PAML v4.4 was used to evaluate each alignment for evidence of positive selection acting on one or more amino acid sites throughout the evolutionary tree of primates. Specifically, we first used codeml model M0 to infer branch lengths for the evolutionary tree corresponding to each alignment, and then we used those branch lengths as input to models M7 and M8 in order to perform a likelihood ratio test (LRT) for evidence of localized positive selection [51]. P-values were calculated by comparing the LRT statistic to the chi-squared distribution with 2 degrees of freedom, and the set of raw p-values was corrected for multiple testing using the Benjamini-Hochberg method [52]. The adjusted p-value for a given alignment represents the overall expected false discovery rate when using that alignment's LRT statistic as a cutoff threshold. Raw and adjusted p-values for the candidate genes are included in Table S1.

### Statistics

The analysis of the differences in distributions used permutation test (n = 100,000) with Kolmogorov-Smirnov statistics. Probability density functions for the various gene sets (Figure 1) were plotted assuming continuous dN/dS values using kernel density estimates. Statistical analyses and graphical representations were performed by using the R package (www.r-project.org). Correlation analysis was performed using Pearson r evaluation.

### Expression vectors

Bi-cistronic pCG expression vectors containing the immediate early promoter of human cytomegalovirus (CMV) coexpressing a candidate gene and BFP via an internal ribosome entry site (IRES) were generated as described elsewhere [53,54]. Briefly, the candidates were amplified from human cDNA obtained from primary blood cells by standard PCR reaction using primers introducing flanking *XbaI* and *MluI* restriction sites for cloning into the expression vector. All PCR-derived inserts were sequenced to confirm their accuracy. Silent mutations were accepted.

### Proviral HIV-1 constructs

Generation of wt HIV-1 (NL4-3-based) proviral reporter constructs containing a functional *nef* gene followed by an IRES and the *eGFP* gene and the triple mutant (Δ*vpu*Δ*nef*Δ*vpr*) have been described previously [19,54,55].

### Cells and transfection

HEK293T cells were maintained in Dulbecco modified Eagle medium (DMEM) supplemented with 10% FCS (1% Glutamine, 1% Penicillin/Streptomycin). A total of 500,000 293T cells per well were seeded in 6-well plates and transfected with 5 μg DNA (1:1 ratio provirus:candidate gene) at a confluence of about 70% by the calcium phosphate method. TZM-bl cells were kindly provided by Drs. Kappes and Wu and Tranzyme Inc. through the NIH AIDS Reagent Program and were kept in DMEM supplemented with 10% FCS (1% Glutamine and 1% Penicillin/Streptomycin). TZM-bl cells express large amounts of CD4, CCR5 and CXCR4 and contain the β-galactosidase gene under the control of the HIV-1 promoter.

### Infectivity assay

The yield of infectious HIV-1 was determined by a 96-well infection assay using TZM-bl indicator cells. Briefly, 6,000 cells were seeded out in 96-well dishes in a volume of 100 μl and infected in triplicate after overnight incubation with 10 or 100 μl of virus stocks. Three days later infection was detected using a galactosidase screen kit from Applied Bioscience as recommended by the manufacturer. β-Galactosidase activities were quantified as relative light units per second (RLU/s) using an Orion II Microplate Luminometer (Berthold).

### p24 antigen ELISA

Nunc immuno maxi sorb surface 96-well plates were coated with a mouse anti-p24 monoclonal (MAK183) antibody (EXBIO) overnight. After blocking, Triton X-100 lysed supernatants or cells were transferred to the 96-well plates. The next day, plates were washed and incubated with a polyclonal rabbit anti-HIV-1 p24 antibody (Eurogentec) for 1 h. Next, plates were washed and incubated with a goat anti-rabbit antibody conjugated with horseradish peroxidase (Dianova, 111-035-008) followed by the addition of TMB peroxidase substrate. The reaction was stopped with 0.5 M H_2_SO_4_ and OD was measured at 450/650 nm.

### Flow cytometric analysis

Flow cytometry analysis of BFP and eGFP reporter expression in 293T cells transfected with bi-cistronic vectors coexpressing the gene candidate and BFP together with proviral HIV-1 IRES eGFP was performed on a FACS CANTO II (BD) flow cytometer essentially as described previously [54]. For analysis, the percentage of BFP/eGFP double positive cells was determined in relation to BFP positive cells in total. All values were normalized to the sample transfected with a control plasmid coding for no functional protein followed by an IRES BFP cassette.

### Cytotoxicity assay

The potential cytotoxicity of candidate genes was evaluated by MTT assay. 293T cells (n=20,000) were transfected in 96-well format with vectors expressing the candidate genes. After 2 days 10 μl of a 5 mg/ml MTT (3-(4,5-dimethylthiazole-2-yl)-2,5-diphenyl tetrazolium bromide, Sigma #M2003) solution were added and incubated for three hours. Thereafter cell free supernatant was discarded, formazan crystals were solubilized in 100 μl DMSO-Ethanol (1:1) and OD was detected at 490/650 nm to evaluate cell viability.

### Promoter reporter assays

To determine the modulation of viral promoter activity, 293T cells were cotransfected with equal amounts (2.5 µg) of firefly luciferase reporter vectors for the HIV-1 long terminal repeat (LTR), HSV thymidine kinase (TK) and CMV immediate early (IE) promoters as well as vectors coexpressing the candidate genes and BFP. The HIV-1 LTR promoter was activated by cotransfection of a plasmid (0.5 µg) expressing HIV-1 NL4-3 Tat if indicated. 40 h post transfection, cells were lysed in Luciferase Cell Culture Lysis Reagent (Promega, E1531) and firefly luciferase activity was determined using the Promega Luciferase Assay System (E1501) according to the manufacturer’s protocol. The pCMV-IE and pHSV-TK red firefly luciferase reporter vectors were purchased from Thermo Scientific (16156 and 16157, respectively). The 5’-LTR of HIV-1 NL4-3 was inserted into the pGL3 enhancer vector from Promega (E1771) via XhoI/MluI. HIV-1 NL4-3 Tat was cloned into a pCG expression vector via XbaI/MluI.

### RNAseq Analysis

Resting CD4+ T cells were purified from two healthy blood donors by Ficoll gradient separation followed by negative selection and magnetic separation using the human CD4+ T Cell enrichment kit supplemented with anti-HLA-DR, anti-CD25 and anti-CD69 (Stem Cell Technologies). Cells (10^6^) were incubated for 24 hours, either with medium only, or with 100 or 1000 IU/ml interferon α (Roferon-A, Roche) or interferon γ (R&D Systems), or with 2 μg anti-CD3/1 μg anti-CD28 and 100 U/ml recombinant interleukin-2 (R&D Systems) to mimic TCR stimulation. After 24 hour incubation, total RNA extraction was performed using Illustra RNAspin mini isolation kit (GE Healthcare) and further processed for mRNA-Seq library preparation (TruSeq RNA sample prep kit, Illumina). 100 bp paired-end sequencing was performed using Illumina HiSeq2000. About 140 mio read pairs were obtained for the samples. Sequencing reads were quality filtered before alignment using Cutadapt [56] and PrInseq [57]. Cleaned sequencing reads were aligned to a build genome using SOAPsplice. Gene expression was assessed by determining the number of mapped reads per gene using HTSeq-count tool, followed by DESeq R package normalization. An additional normalization step taking into account the gene coding length was performed so that the resulting counts could be compared across samples for a given gene, and also between genes.

### Ranking screen

A set of 15,052 protein-coding genes was annotated with nine parameters informative for response to HIV-1 infection or evolutionary history. Parameters included *in vivo* response to HIV-1 infection (i.e. expression correlation with viral load [3], primate dN/dS value, codon-specific positive selection (PAML), measurements of cross species adaptive evolution (the McDonald-Kreitman value comparing human sequence to chimp, orangutan or rhesus [58], burden of synonymous, missense and non-sense variation, intolerance to functional variation (rare variant intolerance score obtained from [59]) and number of paralogs. Burden of synonymous, missense and non-sense variation was taken from exome sequence data on ~6,300 individuals sequenced as part of the NHLBI exome sequencing project (http://evs.gs.washington.edu/EVS/). Burden was calculated per gene as the total number of variants in each class divided by gene length. Genes were ranked for each parameter (with top ranked genes showing high positive correlation with HIV viral load and high levels of within and between species diversity) and the ranks were summed giving equal weight to each measurement. Beginning with all measurements, parameters were iteratively dropped and re-included (individually and in combination) in the ranking and the final model was selected based on the set of parameters that optimized the ranking of known HIV-1 restriction factors. The final set of parameters included *in vivo* response to HIV-1 infection, dN/dS value and evidence for codon-specific positive selection (PAML P-value).

## Additional files

Additional file 1: Table S1. Genes selected on the basis of evolutionary history in primates, expression during HIV-1 infection *in vivo*, or issued from the HIV-1 interaction database. Because of errors in sequencing, annotation and alignment can lead to excessive false positives in downstream evolutionary analyses. Therefore, we assessed dN/dS estimates using both Ensembl Compara’s protein-based alignments (columns c_m0 and c_m), and DNA-based alignments of primate sequences generated from genomic DNA alignments (column g_m0). The “m0” columns (c_m0 and g_m0) show gene-wide dN/dS values estimated using the SLR software under a one-ratio model (i.e. one dN/dS value per gene), while the “m” column (c_m) shows the mean across the gene of all codon-specific dN/dS estimates using SLR's sitewise model (Massingham and Goldman, 2005). The raw and adjusted p-values for sitewise positive selection, measured using the so-called 'site' test for positive selection (Yang, 2007), are included (column paml_pval, and Benjamini-Hochberg FDR-adjusted: paml_adj_pval). In column “Association”, E=differentially expressed during HIV-1 infection *in vivo,* I= included in the NCBI HIV-1. interaction database. The effect of the candidate genes on NL4-3 and D3 HIV-1 is indicated in the following columns. Each value represents the average values (n≥3) obtained in the presence of one of the 27 individual candidates analyzed. Additional file 2: Figure S1. Assessment of possible cytotoxic effects of candidate genes. 293T cells were transfected with 2.5 µg of constructs expressing the candidate genes and the cell viability was examined two days later by MTT assay. Lack of cytotoxicity was confirmed in the CellTiter-Glo® Luminescent Cell Viability Assay. All data were normalized to the BFP only control construct (100%) and are presented as mean ±SD (*n* = 3). **Figure S2.** Assessment of antiviral effects of candidate genes. Impact of individual candidate factors on HIV-1 gene expression, release and infectivity. Candidates were ranked from the left to the right based on their inhibitory effect on the production of infectious wt HIV‑1 (blue). Results obtained for the D3 virus (red) are shown for comparison. The detection limit for CA and CF p24 antigen in the ELISA assay is about 0.5%. **Figure S3.** Correlation between the effect of the candidate genes on wt and D3 HIV-1 gene expression, virus release and viral infectivity. (A) Correlation between the effect of the candidate genes on wt and D3 HIV-1 in the indicated assays. Each symbol represents the average values (n≥3) obtained in the presence of one of the 28 individual candidate genes analyzed. Candidate genes that reduced the values for both wt and D3 virus below 50% (FACS-based assays) or below 10% (p24 expression and infectious virus production) are color coded green; those that achieved this only for the D3 construct yellow; and enhancers of both viruses red. (B) Correlation between the effects of the candidate genes in the different assays for viral gene expression, p24 antigen production and viral infectivity. The upper panels give the results obtained for the wt HIV-1 construct and the lower panel provides the data generated using the D3 mutant construct. Additional file 3: Table S2. Ranking of candidates in a relaxed screen. A set of 15,052 protein-coding genes was annotated with nine parameters informative for response to HIV-1 infection or evolutionary history. Parameters included in vivo response to HIV-1 infection, dNdS value, codon-specific positive selection, measurements of cross species adaptive evolution, burden of synonymous, missense and non-sense variation, intolerance to functional variation and number of paralogs. We highlight in yellow genes selected in the main screen, and in blue, pardigmatic restriction factors.
