# Supplementary material for: Identification of potential HIV restriction factors by combining evolutionary genomic signatures with functional analyses
Source: Retrovirology. 2015 May 16;12:41. doi: 10.1186/s12977-015-0165-5 (PMC4434878; doi:10.1186/s12977-015-0165-5)
Supplement: Additional file 2: Figure S1. — Assessment of possible cytotoxic effects of candidate genes. 293T cells were transfected with 2.5 µg of constructs expressing the candidate genes and the cell viability was examined two days later by MTT assay. Lack of cytotoxicity was confirmed in the CellTiter-Glo® Luminescent Cell Viability Assay. All data were normalized to the BFP only control construct (100%) and are presented as mean ±SD (n = 3). Figure S2. Assessment of antiviral effects of candidate genes. Impact of individual candidate factors on HIV-1 gene expression, release and infectivity. Candidates were ranked from the left to the right based on their inhibitory effect on the production of infectious wt HIV‑1 (blue). Results obtained for the D3 virus (red) are shown for comparison. The detection limit for CA and CF p24 antigen in the ELISA assay is about 0.5%. Figure S3. Correlation between the effect of the candidate genes on wt and D3 HIV-1 gene expression, virus release and viral infectivity. (A) Correlation between the effect of the candidate genes on wt and D3 HIV-1 in the indicated assays. Each symbol represents the average values (n≥3) obtained in the presence of one of the 28 individual candidate genes analyzed. Candidate genes that reduced the values for both wt and D3 virus below 50% (FACS-based assays) or below 10% (p24 expression and infectious virus production) are color coded green; those that achieved this only for the D3 construct yellow; and enhancers of both viruses red. (B) Correlation between the effects of the candidate genes in the different assays for viral gene expression, p24 antigen production and viral infectivity. The upper panels give the results obtained for the wt HIV-1 construct and the lower panel provides the data generated using the D3 mutant construct. [file 12977_2015_165_MOESM2_ESM.docx]

**Supplementary materials**

**Identification of potential HIV restriction factors by combining evolutionary genomic signatures with functional analyses**

Paul J. McLaren, Ali Gawanbacht, Nitisha Pyndiah, Christian Krapp, Dominik Hotter, Silvia F. Kluge, Nicola Götz, Jessica Heilmann, Katharina Mack, Daniel Sauter, Danielle Thompson, Jérémie Perreaud, Antonio Rausell, Miguel Munoz, Angela Ciuffi, Frank Kirchhoff, Amalio Telenti

**Supplemental Materials**

**Table S1. Genes selected on the basis of evolutionary history in primates, expression during HIV-1 infection *in vivo,* or issued from the HIV-1 interaction database.** To investigate in detail the recent molecular evolution of the 57 candidate genes, we collected all available orthologous primate transcripts for each human gene from version 62 of the Ensembl Compara database. Sequences were then filtered for low-quality genomic material using sequence quality scores downloaded from the genomic sequencing institution where available, masking any codons containing nucleotides with a quality scores of less than 30 (corresponding to an expected error rate of 1 error per 1,000 bases) with 'NNN'. Each set of orthologous transcripts was then aligned with PRANK's codon model, which was shown to be an effective and conservative aligner when used in conjunction with PAML [[1](#_ENREF_1)]. To avoid errors resulting from local misalignment, annotation or assembly errors, we used PAML to infer substitution events and identify 15-amino acid windows with very high rates of apparent substitution and masked those regions with Ns. In addition, we tested a paralog filter in order to avoid estimating dN/dS based on multiple duplicated copies of a gene from one species (paralogous genes are expected to have undergone more adaptive change). The paralog filter kept the copy most similar to the human sequence. We found that the conservative sequence and alignment filtering steps applied were important to minimize the possibility that sequencing, assembly, and alignment errors – which are expected to be more prominent in non-human primate genomes – would cause the alignment of non-homologous primate codons and subsequent false positives in the sitewise detection of positive selection.

Because of errors in sequencing, annotation and alignment can lead to excessive false positives in downstream evolutionary analyses. Therefore, we assessed dN/dS estimates using both Ensembl Compara’s protein-based alignments (columns c_m0 and c_m), and DNA-based alignments of primate sequences generated from genomic DNA alignments (column g_m0). The “m0” columns (c_m0 and g_m0) show gene-wide dN/dS values estimated using the SLR software under a one-ratio model (i.e. one dN/dS value per gene), while the “m” column (c_m) shows the mean across the gene of all codon-specific dN/dS estimates using SLR's sitewise model [[2](#_ENREF_2)]. The raw and adjusted p-values for sitewise positive selection, measured using the so-called 'site' test for positive selection [[3](#_ENREF_3)] are included (column paml_pval, and Benjamini-Hochberg FDR-adjusted: paml_adj_pval). In column “Association”, E=differentially expressed during HIV-1 infection *in vivo*, I= included in the NCBI HIV-1 interaction database. The effect of the candidate genes on NL4-3 and Δ3 HIV-1 is indicated in the following columns. Each value represents the average values (n≥3) obtained in the presence of one of the 27 individual candidates analyzed.

1. Fletcher W, Yang Z: **The effect of insertions, deletions, and alignment errors on the branch-site test of positive selection.** *Mol Biol Evol* 2010, **27:**2257-2267.

2. Massingham T, Goldman N: **Detecting amino acid sites under positive selection and purifying selection.** *Genetics* 2005, **169:**1753-1762.

3. Yang Z: **PAML 4: phylogenetic analysis by maximum likelihood.** *Mol Biol Evol* 2007, **24:**1586-1591.

**Table S2. Ranking of candidates in a relaxed screen.** A set of 15,052 protein-coding genes was annotated with nine parameters informative for response to HIV-1 infection or evolutionary history. Parameters included in vivo response to HIV-1 infection, dNdS value, codon-specific positive selection, measurements of cross species adaptive evolution, burden of synonymous, missense and non-sense variation, intolerance to functional variation and number of paralogs. We highlight in yellow genes selected in the main screen, and in blue, paradigmatic restriction factors.

**
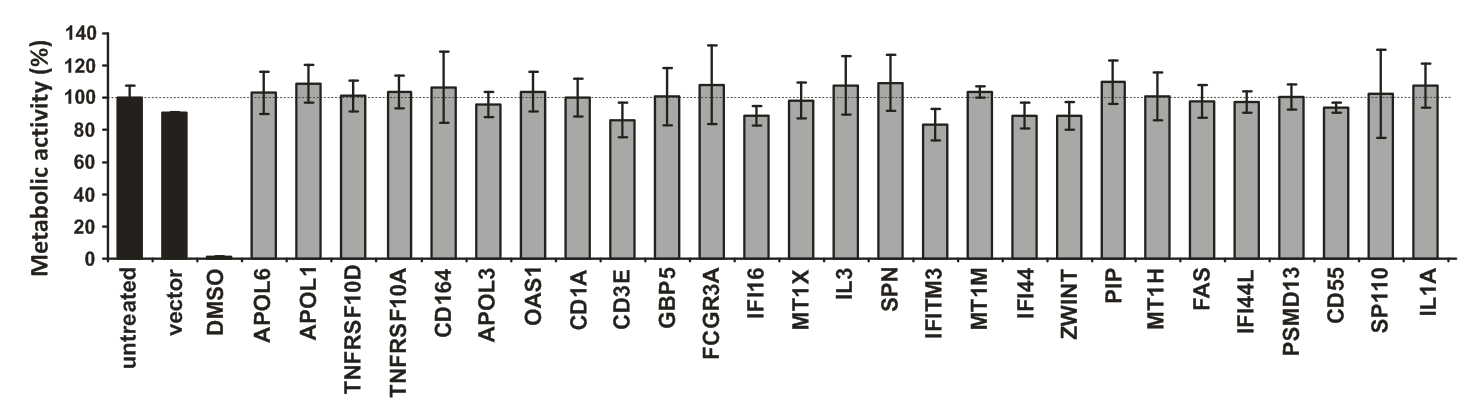
Figure S1. Assessment of possible cytotoxic effects of candidate genes.** 293T cells were transfected with 2.5 µg of constructs expressing the candidate genes and the cell viability was examined two days later by MTT assay. Lack of cytotoxicity was confirmed in the CellTiter-Glo® Luminescent Cell Viability Assay. All data were normalized to the BFP only control construct (100%) and are presented as mean ±SD (*n* = 3).


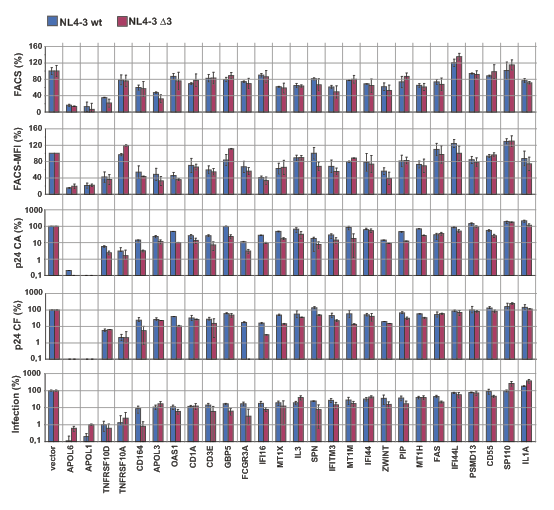


**Figure S2. Assessment of antiviral effects of candidate genes.** Impact of individual candidate factors on HIV-1 gene expression, release and infectivity. Candidates were ranked from the left to the right based on their inhibitory effect on the production of infectious wt HIV‑1 (blue). Results obtained for the Δ3 virus (red) are shown for comparison. The detection limit for CA and CF p24 antigen in the ELISA assay is about 0.5%.

**
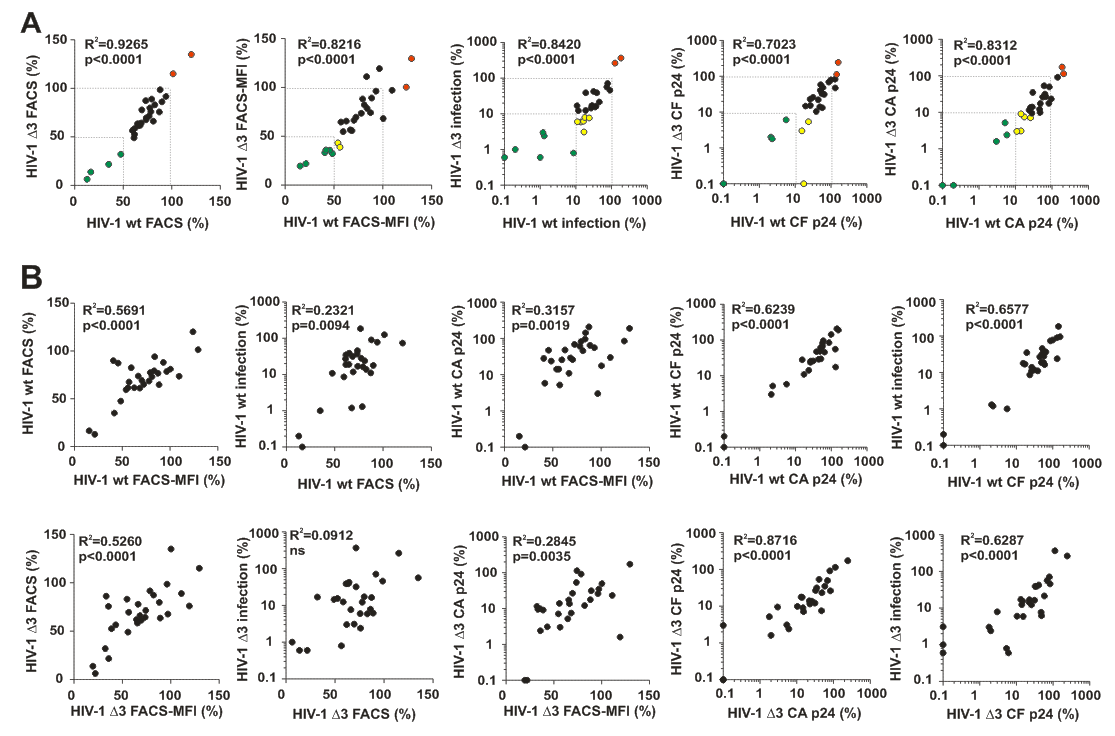
**

**Figure S3. Correlation between the effect of the candidate genes on wt and Δ3 HIV-1 gene expression, virus release and viral infectivity.** (A) Correlation between the effect of the candidate genes on wt and Δ3 HIV-1 in the indicated assays. Each symbol represents the average values (n≥3) obtained in the presence of one of the 28 individual candidate genes analyzed. Candidate genes that reduced the values for both wt and Δ3 virus below 50% (FACS-based assays) or below 10% (p24 expression and infectious virus production) are color coded green; those that achieved this only for the Δ3 construct yellow; and enhancers of both viruses red. (B) Correlation between the effects of the candidate genes in the different assays for viral gene expression, p24 antigen production and viral infectivity. The upper panels give the results obtained for the wt HIV-1 construct and the lower panel provides the data generated using the Δ3 mutant construct.
